# Turkish adaptation and validation of revised Hospital Survey on Patient Safety Culture (TR – HSOPSC 2.0)

**DOI:** 10.1186/s12912-022-01112-9

**Published:** 2022-11-25

**Authors:** Emel Filiz, Müjdat Yeşildal

**Affiliations:** Department of Healthcare Management, Faculty of Health Sciences, Selcuk University, Selçuklu, 42150 Konya, Turkey

**Keywords:** Safety culture, Psychometric, Validity, Reliability, Turkish

## Abstract

**Aim:**

This study is aimed to determine the validity and reliability of the Turkish adaptation of the HSOPSC 2.0 for Turkish hospitals.

**Methods:**

This two-stage psychometric study was conducted with 613 nurses (The response rate of the nurses participated in the study is 72.11%.) at a university hospital between July 2021 and February 2022. In the first stage, the adaptation of the scale was performed. Construct validity was determined at the second stage using confirmatory factor analysis. Reliability was tested using the internal consistency coefficient.

**Results:**

The adaptation results showed that the Turkish version of the scale was adequate for language and content validation. This scale, consisting of 32 items and ten subscales, showed a significantly good fit with the original scale according to confirmatory factor analysis. The Cronbach's alpha coefficient for the subscales ranged between 0.72 and 0.82.

**Conclusions:**

The Turkish version of the Hospital Survey on Patient Safety Culture consistently showed acceptable psychometric reliability and validity characteristics.

## Background

Today, Patient Safety is recognized as the basic principle of healthcare. Many high-income countries published studies indicating that many patients are diversely harmed during healthcare delivery [1]. One in every ten patients in high-income countries is harmed while receiving hospital care [2], and 2.6 million people die every year due to unsafe care in hospitals in low and middle-income countries [3].

Patient safety in healthcare organizations has received much attention following the Institute of Medicine (IOM) report called ‘To Error is Human.’ The report emphasized the importance of significantly creating a safety culture in healthcare institutions to improve patient safety and quality of care. The IOM states that when there is a safety culture where adverse events can be reported without people being blamed, they have the opportunity to learn from their mistakes, and that it is possible to make improvements to prevent future human and system errors to enable the improvement of patient safety [4]. AHRQ defines the safety culture as follows: “*The safety culture of an organization is the product of individual and group values, attitudes, perceptions, competencies, and patterns of behavior that determine the commitment to, and the style and proficiency of, an organization’s health and safety management.”* [5].

The role of culture in improving the safety of patients and healthcare professionals is becoming increasingly important. The relationship between the high level of safety culture and reduced length of hospital stay, readmissions, and medication errors has been emphasized [6]. Fewer adverse events are reported in nursing units that adopt a positive safety culture. In a systematic review conducted on the relationship between safety attitudes of nurses at acute care hospitals and patient outcomes, it was found that those who had positive safety attitudes reported fewer patient falls, medication errors, pressure injuries, healthcare-related infections, and deaths. In addition, in this study, it was concluded that a positive safety culture affects patient satisfaction. It was also determined that effective teamwork decreases adverse patient outcomes [7].

The World Health Organization (WHO) calls on countries to create a safety culture in their healthcare systems in Global Patient Safety Action Plan 2021–2030. It recommends countries conduct regular surveys of the safety culture in healthcare organizations and contribute to international benchmarking studies. Therefore, healthcare systems will be able to observe their strengths and weaknesses related to safety culture and utilize the experience of other countries to improve patient safety while reducing unwanted events [8]. There are many measurement instruments developed to measure the safety culture perceptions of healthcare professionals, which are being used increasingly around the world [9]. Some of these measurement instruments were developed to be used in specific care units such as operating rooms and geriatric care units [10, 11], while others are used for an overall assessment [12–17]. The instruments differ in terms of psychometric properties, number of subscales and items (Table 1). The majority consists of subscales such as “management support”, “safety systems”, “safety attitudes of staff”, “reporting incidents” “opennes to communication” “organizational learning” and “teamwork” [18]. Safety Attitude Questionnaire (SAQ) and the HSOPSC 1.0 are two of the most reliable and popular instruments among those of which reliability and validity studies were conducted [9, 18]. Since these two scales, which include fundamental dimensions of patient safety, are translated into various languages and have been used for a long period of time, they also provide benchmarking data. The HSOPSC version of 1.0 consists of 42 items and 12 subscales. The factor loading of the survey was found to range between 0.36 and 1.00 in psychometric assessment. Reliability of the each of 12 subscales of the survey was assessed with confirmatory factor analysis. Cronbach’s alpha was found to range between ≥ 60 and (62–84), which indicate an acceptable level of reliability. The survey was not designed solely for a specific unit or professional group, instead, it can be applied to all employees working in all units of the hospital and healthcare services. The Turkish adaptation of the HSOPSC was studied by Bodur and Filiz [19], and was used in several studies conducted in Turkey [20–25]. These studies contribute to reveal challenging issues about patient safety in hospitals, as well as to make both national and international comparisons. In 2019, AHRQ released a new version of the survey under the name of HSOPSC 2.0. Although the first version of the survey is still in use, AHRQ is now encouraging the use of the name HSOPSC 2.0 [17]. The HSOPSC 2.0 has fewer items, and the subscales have been updated to reflect the content of the included items [17]. It is important that PSC assessments to be conducted in the future at our hospitals can be compared with other cultures once use of the new version become widespread. In this study, it was aimed to determine the validity and reliability of the Turkish version of the HSOPS 2.0.

**Table 1:** Examples of widely used Patient Safety Culture measurement instruments and relevant characteristics

Instrument	Subscale/Item	Reliability
Hospital Survey on Patient Safety Culture 1.0 (HSOPSC 1.0)	12/42	α ≥ 70 (except Staffing (staffing = 62))
Safety Attitudes Questionnaire SAQ (Sexton 2006)	6/60	Raykov ṅ Coefficient = 0.90
Patient Safety Climate in Healthcare Organisations PSCHO (Singer 2007)	9/38	0.50–0.89
Short-form Patient Safety Climate in Healthcare Organisations (Benzer 2017)	3/15	0.74–0.84
Patient and Occupational Safety Culture Questionnaire (Wagner 2018)	23/73	0.59–0.89

## Methods

### Study design and participants

This methodological research was carried out in internal, surgical, and intensive care units of a 1350-bed university hospital in Konya province, located in the Central Anatolia Region of Turkey. A total of 970 nurses work at the subject hospital. Of these, 850 nurses who contacted patients were invited to the study. The research sample was calculated since the sample size should be at least five or ten times the number of items in validity and reliability studies [26]. The data were collected from 613 nurses using the convenience sampling method. 72.11% of nurses who were invited to the study were reached. Inclusion criteria were determined as working as a nurse for at least one year in clinics offering direct patient care. Since nursing turnover rates are high at the Faculty of Medicine Hospital, those who had been working for at least one year were included. In addition, nurses working in the managerial positions and those who were on leave were not included.

### Data collection

The study data were collected online through Google Forms due to the COVID-19 pandemic between July 2021 and February 2022. After obtaining the ethics committee’s approval and institutional permission, the survey (via emailing Google Forms link) was sent to the nurses through the hospital quality management unit. The survey includes the informed consent form, the demographic data form, and the Hospital Survey on Patient Safety Culture (TR—HSOPSC 2.0). It takes approximately 10–15 min to complete the survey.

### Instruments

#### Personal information form

The form consists of four questions related to the demographic and occupational characteristics of the nurses, such as the unit they work in, duration of their experience in the current hospital and the current unit, and weekly working hours.

#### Hospital Survey on Patient Safety Culture (HSOPSC 2.0)

The HSOPSC 2.0 survey is a revised version of the HSOPSC 1.0 which was designed based on feedback and suggestions from users and stakeholders [17]. The number of items was reduced from 42 to 32, and the number of subscales decreased from 12 to 10. Overall Perceptions of Patient Safety and Teamwork Across Units subscales were removed in the revised version. The majority of the remaining items in the survey were re-evaluated. HSOPSC 2.0 was published in 2019 due to the first pilot implementation at 44 hospitals in 2017 and 25 hospitals in 2019. The Cronbach's alpha coefficients of the subscales of the HSOPSC 2.0 ranged between 0.67 and 0.89 in the USA study. This survey can be applied to all personnel from cleaning workers and security members to nurses and hospital managers, or solely to one specific unit or personnel group [17].

The 32 safety culture items in the HSOPSC 2.0 are measured on 5-point response scales in terms of agreement (strongly disagree to strongly agree) or frequency (never to always), as well as an option for “does not apply or “do not know.” Two single items ask respondents (1) to provide an overall rating of patient safety for their unit (i.e., a patient safety grade) using a 5-point response scale (poor to excellent), and (2) how many patient safety events they have reported.

#### Translation and cultural adaptation of the instrument

The translation process was performed after receiving permission from AHRQ via email. The recommended steps were followed to achieve language and cross-cultural equivalence. The application of this method includes the steps of (1) forward translation, (2) synthesis of translations (3) reverse translation, (4) expert panel, (5) cognitive interview—pre-test and (6) final version [27–29].

Two academic nurses, two nurses who work in the hospital and have a postgraduate degree, and two specialist physicians were included in the translation committee. They were native Turkish speakers with fluent English. In the first stage, the translations of the questionnaire into Turkish were performed separately by the committee members. In the second stage, the translations were collected, and synthesized by researchers and an expert fluent in both languages. At this stage, inconsistencies and errors in the translation were corrected, and the first version of the Turkish adaptation was obtained. In the third stage, the Turkish text was translated separately by 2 translation experts who were native speakers of English with fluent Turkish, and had not seen the original version of the questionnaire. In the fourth stage, the original version, Turkish adaptation and retranslation into the original language were evaluated by a 10-person expert panel consisting of academic and clinical patient safety experts. The purpose of this phase was to detect insufficient expressions in the translation and inconsistencies between the two languages ​​and to evaluate them in terms of face validity and content validity. Using a 4-point scale ranging from 1 (inappropriate) to 4 (very appropriate), the experts rated the cultural relevance and consistency of each item. During the Pilot Implementation and Cognitive Review stage, items were presented to 10 nurses (with a master's degree) working in internal-surgical units for evaluation. Nurses were asked to make suggestions for expressions that were not understood. They were also encouraged to suggest alternative ways of expressing the meaning of the original items. Minor changes were made in accordance with the suggestions and comments of the participants, and the Turkish version of the HSOPSC 2.0 questionnaire, TR – HSOPSC 2.0, was created.

### Ethical consideration

Permission for the Turkish adaptation was obtained via email from the Agency for Healthcare Research and Quality (AHRQ), which developed the original survey. Before the study, institutional permission and ethics committee approval (Date: 15.04.2020; Decision no: 2020/481) were obtained. Once nurses were given written information about the study, those who agreed to participate completed the forms and surveys after reading the consent form and checking the “I Agree to Participate in the Study” box.

### Data analysis

The data were analyzed using SPSS (Statistical Package for Social Sciences) for Windows 26.0 and AMOS (Analysis of Moment Structures) 25.0 programs. Descriptive statistics (number. percentage, minimum and maximum, mean, and standard deviation) were used for data evaluation. The internal consistency was evaluated using the Cronbach's alpha and item-total score correlation. For factor loads, the cut-off was set at > 0.50. For validity analyses, exploratory factor analysis, discriminant analysis and confirmatory factor analysis were used. X^2^/df, RMSEA, SRMR, TLI, and GFI were used for goodness-of-fit values. The data were found to show normal distribution according to the Kolmogorov–Smirnov test. Statistical significance was set at 0.05.

## Results

### Participants’ characteristics

As seen in Table 2, of the 613 nurses participating in the study, 36.1% work in internal units, and 34.9% work in intensive care. The weekly working hour of nurses was 42.50 h (SD = 4.75), the duration of experience in the current hospital was 10.24 years (SD = 7.33), and the time of experience in the current unit was 7.52 years (SD = 6.31).

**Table 2:** Demographic Characteristics of Study Participants (*N* = 613)

Characteristics	N	Percent	M	SD
Surgical units	178	29.0		
Internal units	221	36.1		
Intensive care	214	34.9		
Years in current hospital			10.24	7.33
Years in the current unit			7.52	6.31
Weekly working hour			42.50	4.75

### Validity analysis

The results of the expert panel review were used to conclude the content of the TR – HSOPSC 2.0, and to evaluate the face validity and content validity. The expert panel suggested minor revisions to improve the readability and intelligibility of the items in terms of face validity. In the next step, the content validity of the adapted TR—HSOPSC 2.0 was calculated for each item and subscale by evaluating the items using the content validity index (CVI). Lawshe's method was used to calculate the CVI [30]. As a result of the analysis, it was determined that the CVI, which was 0.96 for the overall scale, ranged between 0.90 and 0.98 for the items. The significance of the content validity index was above 0.80 [31, 32], which was considered acceptable.

Prior to the factor analysis, the suitability of the data set for factor analysis was evaluated using KMO and Bartlett's test for construct validity. The KMO sample adequacy test result was found to be at a sufficient level as 0.86. Bartlett's test result was found to be significant (*p* < 0.001). The 10-factor structure with an eigenvalue greater than 1 was explained with a total variance of 71.8%. As a result of the EFA, the rotated factor loads were examined using the Varimax rotation technique.

The factor loads of the TR – HSOPSC 2.0 range between 0.58 and 0.80. The first factor Teamwork is between 0.65 and 0.74, the second factor Staffing and Work Pace is between 0.70 and 0.76, the third factor Organizational Learning – Continuous Improvement is between 0.62 and 0.64, the fourth factor Response to Error is between 0.65 and 0.70, the fifth factor Supervisor, Manager, or Clinical Leader Support for Patient Safety is between 0.72 and 0.76, the sixth factor Communication about Error is between 0.63 and 0.72, the seventh factor Communication Openness is between 0.69 and 0.76, the eighth factor Reporting Patient Safety Events is between 0.59 and 0.63, the ninth factor Hospital Management Support for Patient Safety is between 0.58 and 0.66, and the tenth factor Handoffs and Information Exchange is between 0.73 – 0.80.

As seen in Table 3, the highest average scores of the TR – HSOPSC 2.0 were 3.73, obtained from Reporting Patient Safety Events, and 3.43, obtained from Staffing and Work Pace subscale. In contrast, the lowest average score was 2.24, obtained from the Communication about Error subscale, followed by 2.62, obtained from Hospital Management Support for Patient Safety.

**Table 3:** Descriptive statistics, reliability coefficients, and factor loads of the TR – HSOPSC 2.0 (*N* = 613)

Subscale	Item	M ± SD	Factor loads	Cronbach's alpha of the item Deleted	Cronbach's alpha
**Teamwork**		3.20 ± 0.58			0.75
	A1	3.13 ± 1.16	0.65	0.73	
	A8	2.36 ± 1.07	0.72	0.72	
	A9^a^	4.10 ± 1.01	0.74	0.74	
**Staffing and Work Pace**		3.43 ± 0.52			0.73
	A2	2.94 ± 1.23	0.70	0.71	
	A3	3.23 ± 1.20	0.71	0.72	
	A5^a^	4.03 ± 1.03	0.76	0.72	
	A11^a^	3.52 ± 1.08	0.74	0.71	
**Organizational Learning – Continuous Improvement**		2.97 ± 0.55			0.74
	A4	2.78 ± 1.19	0.62	0.71	
	A12	3.16 ± 1.17	0.63	0.68	
	A14^a^	2.98 ± 1.15	0.64	0.72	
**Response to Error**		3.22 ± 0.41			0.81
	A6^a^	3.63 ± 1.19	0.65	0.75	
	A7^a^	4.14 ± 0.96	0.66	0.74	
	A10	2.44 ± 1.11	0.68	0.73	
	A13^a^	2.68 ± 1.13	0.70	0.72	
**Supervisor, Manager, or Clinical Leader Support for Patient Safety**		2.62 ± 0.54			0.77
	B1	2.77 ± 1.20	0.72	0.73	
	B2^a^	3.22 ± 1.19	0.76	0.72	
	B3	1.86 ± 0.96	0.74	0.69	
**Communication about Error**		2.24 ± 0.84			0.73
	C1	2.36 ± 1.07	0.63	0.69	
	C2	1.90 ± 1.01	0.68	0.70	
	C3	2.44 ± 1.11	0.72	0.68	
**Communication Openness**		2.83 ± 0.52			0.82
	C4	2.48 ± 1.08	0.76	0.81	
	C5	3.16 ± 1.17	0.70	0.78	
	C6	2.68 ± 1.13	0.69	0.79	
	C7^a^	2.98 ± 1.16	0.71	0.80	
**Reporting Patient Safety Events**		3.73 ± 0.96			0.74
	D1	3.37 ± 1.34	0.59	0.70	
	D2	4.10 ± 1.51	0.63	0.68	
**Hospital Management Support for Patient Safety**		2.62 ± 0.48			0.76
	F1	1.90 ± 1.01	0.66	0.68	
	F2	2.44 ± 1.11	0.71	0.72	
	F3^a^	3.52 ± 1.08	0.58	0.71	
**Handoffs and Information Exchange**		3.06 ± 0.54			0.72
	F4^a^	2.84 ± 1.17	0.80	0.71	
	F5^a^	3.32 ± 1.13	0.73	0.72	
	F6	3.02 ± 1.15	0.79	0.68	

^a^Reverse-coded item

The highest average scores were obtained from item A7 with 4.14: “When an event is reported in this unit, it feels like the person is being written up, not the problem” and item D2 with 4.10: “When a mistake reaches the patient and could have harmed the patient, but did not, how often is this reported?”, while the lowest average scores were obtained from item B3 with 1.86: “My supervisor, manager, or clinical leader takes action to address patient safety concerns that are brought to their attention” and item C2 with 1.90: “When errors happen in this unit, we discuss ways to prevent them from happening again.”

In Table 4, the model fit indices of the TR—HSOPSC 2.0 were given. In confirmatory factor analysis, values of X^2^/ df = 2.86, RMSEA = 0.07, SRMR = 0.05 provided an acceptable fit, while TLI = 0.91 and GFI = 0.93 provided a good fit [33–35]. A good relationship was found between the items and the subscales. Covariance was not performed between the items since the correction indices were not found to be at a significant load.

**Table 4:** Fit indices of the models

Fit indices	Good Fit	Acceptable Fit	Model
X^2^/df	0 ≤ X^2^/df ≤ 2	2 ≤ X^2^/df ≤ 5	2.86
RMSEA	0 ≤ RMSEA ≤ 0.05	0.05 < RMSEA < 0.08	0.07
SRMR	0 ≤ SRMR ≤ 0.05	0.05 < SRMR < 0.08	0.05
TLI	0.90 ≤ TLI ≤ 1	0.85 ≤ TLI < 0.90	0.91
GFI	0.90 ≤ GFI ≤ 1	0.85 ≤ GFI < 0.90	0.93

Discriminant analysis was performed to test the discrimination of the items scoring the highest and lowest at 27%. Accordingly, the Student's t-test compared the lower and higher cut groups. There was a significant difference between the lower scored group ( n = 240), and the higher scored group ( n = 240) ( *p* < 0.001) for each mean item score.

### Internal validity

Descriptive analyses and reliability coefficients of the TR – HSOPSC 2.0 were given in Table 3. Cronbach's alpha coefficients were as follows: 0.75 for Teamwork, 0.73 for Staffing and Work Pace, 0.74 for Organizational Learning—Continuous Improvement, 0.81 for Response to Error, 0.77 for Supervisor, Manager, or Clinical Leader Support for Patient Safety, 0.73 for Communication about Error, 0.82 for Communication Openness, 0.74 for Reporting Patient Safety Events, 0.76 for Hospital Management Support for Patient Safety, and 0.72 for Handoffs and Information Exchange. If the item was deleted, Cronbach's alpha would indicate that the consistency would not be improved by removing any items.

## Discussions

In this study, the validity and reliability of the new version of the HSOPSC was evaluated based on the data obtained from the nurses working in a university hospital. Since national and international data to be compared can be obtained using a valid and reliable instrument in patient safety culture studies, the Turkish adaptation of the scale is of great importance.

The study findings showed good internal consistency, content validity, and construct validity, indicating that the HSOPSC 2.0 can measure nurses' perceptions of patient safety culture in Turkish hospitals. For the 32-item structure of the Turkish version, the CFA results supported the 10-factor structure. In addition, each item provided evidence of the construct validity by contributing to its expected subscale.

The content validity of the TR – HSOPSC 2.0 was evaluated using CVI. According to the CVI results, the equivalence of the items in the Turkish version was assessed. Content validity indices are expected to be above 0.80 [31]. The content validity index was at an acceptable level in the current study. Therefore, no items were removed from the scale regarding the content validity. The construct validity of the scale was evaluated using CFA. CFA is a type of structural equation modeling that reveals the relationships between items and factors [34]. The confirmatory factor analysis results showed that X^2^/df, RMSEA, and SRMR values were acceptable, while TLI and GFI values showed a good fit [36]. There was a good correlation between the items and the subscales. Since correction indices were not found to be at a significant load, covariance was not performed between the items [31]. In addition, discriminant analyses indicated that each item achieved the difference between those who received the highest and lowest scores.

In the 10–factor structure of the TR—HSOPSC 2.0, the Cronbach's alpha ranged from 0.72 to 0.82. The Cronbach’s alpha of the original version of the HSOPSC 2.0 ranges between 0.67 and 0.89, which was found to be ≥ 72 for all composites except Staffing and Work Pace (0.67) [17]. Although the survey has already been translated into various languages and widely used, there are only two published studies which conducted the cultural adaptation of the AHRQ. The Indonesian version of the HSPSC 2.0 was validated by Suryani et al. as 32 items and 10 factors. Factor loads range from 0.47 to 0.65. The Cronbach's alpha value of the composites are ≥ 70 except Communication openness and Response to error (0.67–0.81) [37]. In another study, Lee and Dahinten [38] designed the Korean version of the survey, and they removed one of the items (This unit relies too much on temporary, float, or PRN staff) which was not found to be applicable to the Korean health system. Therefore, the item was validated to consist of 31 items and 10 subscales. The Cronbach’s alpha values of nine composites of the survey ranged between 0,71 and 0,83 except the “Staffing and work pace” (Cronbach’s alpha of 0.61). The current results indicate that psychometric properties of the Turkish version of the HSOPSC 2.0 are at an acceptable level. Since the new version is shorter and easier to understand, it is considered to help create awareness among nurses and other healthcare personnel about PS, as well as determine the relevant deficiencies and conduct internal and external comparisons.

### Limitations

Although this study provides evidence of the reliability and construct validity of the TR—HSOPSC 2.0 to measure the patient safety culture in Turkish hospitals, there are certain limitations. The physicians and other healthcare personnel were not included in the study due to busy working hours during the COVID-19 pandemic. All participants work in a general tertiary hospital affiliated with medical schools. In addition, all participants consist of internal – surgical units and intensive care nurses who provide direct care. The vast majority of nurses are composed of females. Therefore, the results may not represent all Turkish nurses and cannot be generalized to other healthcare professionals.

Additionally, there are very few psychometric studies to discuss this measurement instrument. Consequently, its psychometric properties should be studied within the context of a broader validation in the future.

## Conclusions

As a result of a literature review, this study was the first to examine the psychometric properties of the HSOPSC 2.0 in the context of Turkish healthcare services. The study provides preliminary evidence of the translated and adapted instrument's internal consistency, content validity, and construct validity based on the data obtained from direct care nurses working in medical-surgical units in Turkey. Our CFA results confirm use of the 32-item and 10-factor TR—HSOPSC 2.0. Further research is needed to investigate its psychometric properties for broadly validated implementations.

## Data Availability

The data sets used and analyzed during this study can be provided from the corresponding author on reasonable request.

## Abbreviation

HSOPSC: Hospital Survey on Patient Safety Culture
